# Volatile Organic Compounds, Indole, and Biogenic Amines Assessment in Two Mediterranean Irciniidae (Porifera, Demospongiae)

**DOI:** 10.3390/md19120711

**Published:** 2021-12-17

**Authors:** Antonella Aresta, Pietro Cotugno, Nicoletta De Vietro, Caterina Longo, Maria Mercurio, Pere Ferriol, Carlo Zambonin, Carlotta Nonnis Marzano

**Affiliations:** 1 Department of Chemistry, University of Bari Aldo Moro, 70125 Bari, Italy; antonellamaria.aresta@uniba.it (A.A.); pietro.cotugno@uniba.it (P.C.); nicoletta.devietro@uniba.it (N.D.V.); carlo.zambonin@uniba.it (C.Z.); 2 Department of Biology, University of Bari Aldo Moro, 70125 Bari, Italy; maria.mercurio@uniba.it (M.M.); carlotta.nonnismarzano@uniba.it (C.N.M.); 3 Department of Biology, University of the Balearic Islands, 07122 Palma, Spain; pere.ferriol@uib.es

**Keywords:** VOCs, indole, biogenic amines, Mediterranean Irciniidae, SPME-GC-MS

## Abstract

Solid phase microextraction (SPME) coupled to gas chromatography-mass spectrometry (GC-MS) was employed for the headspace determination of the volatile organic fraction emitted by two of the most common Mediterranean demosponges, *Ircinia variabilis* and *Sarcotragus spinosulus*, and of indole and some biogenic amines released by sponges in an aqueous medium. A total of 50/30 µm divinylbenzene/carboxen/polydimethylsiloxane and 75 µm carboxen/polydimethylsiloxane fibers were used for the headspace extraction of low molecular weight sulfur compounds from a hermetically sealed vial containing sponge fragments, while the direct immersion determination of indole and biogenic amines was performed. The biogenic amines were extracted after in-solution derivatization with isobutyl chloroformate. All analytical parameters (linearity, limits of detection, and quantification, precision, and recovery) were evaluated for indole and biogenic amines. SPME-GC-MS proved to be a reliable means of highlighting the differences between molecules released by different sponges, principally responsible for their smell. The combined approaches allowed the identification of several volatile compounds in the headspace and other molecules released by the sponges in an aqueous medium, including indole and the BAs cadaverine, histamine, isobutylamine, isopentylamine, propylamine, 2-phenylethylamine, putrescine and tryptamine. The results obtained represent a further contribution to the picture of odoriferous molecules secreted by sponges.

## 1. Introduction

Sponges (phylum Porifera) are common and abundant benthic invertebrates colonizing marine and freshwater habitats worldwide [1]. They are active filter-feeders constantly permeated by a water flow running through their aquiferous system, thus, constantly interacting with microorganisms, even through symbiotic relationships [2]. Indeed, sponges host a wide and diverse community of microorganisms, which in some cases represent up to 40% of the sponge wet weight [2]. They are, therefore, considered “holobionts”, complex systems consisting of a eukaryotic host and its microbiota [3].

Sponge holobionts produce a wide range of metabolites that can be used in intercellular communication, or in chemical defense against substrate competitors, predators and pathogens [3,4]. Recent research revealed that about 90% of the sponge species defend themselves from predators by means of deterrent chemicals [5]. At present, poriferans are considered among the most effective producers of biologically active chemicals and represent the central focus of studies for the discovery of new drugs in marine organisms [6,7].

The so-called “horny sponges” belonging to the order Dictyoceratida (Class Demospongiae, Subclass Keratosa), widespread and rather abundant on Mediterranean bottoms, are currently considered the most productive, since they have contributed over 20% of newly discovered sponge-derived bioactive compounds [8]. These compounds are very diverse in both structure and bioactivity, with terpenes representing 73% of the reported chemicals, followed by nitrogenous compounds (13%). Specifically, the family Irciniidae, with the three genera *Ircinia*, *Psammocinia*, and *Sarcotragus*, provided 35 new compounds, mainly showing antibacterial and cytotoxic activity, in the 2013–2019 period alone [9].

The Irciniidae, like other species of sponges, are characterized by an unpleasant odor, clearly perceptible when exposed to the air and even more intense when they are cut. The manipulation of different species of Keratosa during in situ sponge culture experiences [10,11,12,13,14] allowed researchers to perceive the odor released by the sponges and to detect the effect on researchers, due to inhalation or contact with the specimens. Effects such as nausea, headache and skin rashes have sparked our interests in the search for volatile compounds responsible for odor in sponges, bearing in mind their possible biological role. To the best of our knowledge, little research has been carried out to characterize the volatile chemicals produced by marine sponges [15,16,17,18], and those also perceptible in water, whether they are hydrophilic or hydrophobic molecules [19,20], bearing in mind their possible role in the communication within the holobiont and in the defense against external organisms. As regards the genus *Ircinia*, Duque et al. [17] and Pawlik et al. [21] focused on Caribbean species, whereas information on some Irciniidae collected from the Western Mediterranean is available in the grey literature [22].

Among nitrogenous compounds, indole is characterized by a typical fecal odor. It is originated from the amino acid tryptophan by the action of bacteria and is involved in various aspects of bacterial life [23]. Indole alkaloids are marine natural products, commonly found in sponges, that exhibit a wide range of biological activities [24,25]. Finally, biogenic amines (BAs) are ubiquitous substances synthesized by the microbial, plant, and animal metabolism. Many of them are toxic and have an unpleasant smell [26]. The search for BAs in marine organisms is aimed at assessing the risk linked to the consumption of seafood products subjected to bad storage conditions or manufacturing processes [27]. So far, the presence of BAs in relation with the emission of unpleasant odors in sessile marine invertebrates has never been studied.

Solid phase microextraction (SPME) is an effective technique for the extraction of organic compounds from air and water by means of a fiber coated with a selectable polymeric phase. The extraction can be carried out exposing fibers to the headspace (HS-SPME) of solid/liquid matrixes, or by direct immersion (DI-SPME) in a small volume of aqueous samples [28].

In the present research, the SPME technique was applied to the detection of sulfur compounds, indole and some BAs produced by two Irciniidae, namely *Ircinia variabilis* and *Sarcotragus spinosulus*, living along the southern Italian coasts. The hypothesis to test was that these compounds are responsible for the sponges’ odor. HS and DI-SPME were both used to extract the substances secreted by sponges, while gas chromatography-mass spectrometry (GC-MS) was employed for the analytical step.

## 2. Results

Volatile organic compounds (VOCs) extraction from the headspace (HS) of a vial containing fragments of sponges was performed using four SPME fibers, namely carbowax-polyethylene glycol (PEG), polyacrylate (PA), divinylbenzene/carboxen/polydimethylsiloxane (DVB/CAR/PDMS), and carboxen/polydimethylsiloxane (CAR/PDMS). The obtained SPME-GC-MS chromatographic profiles were always very complex and with a certain variability between species (Figure S1). Among the volatile compounds extracted by the different fibers, species belonging to various chemical classes could be attributed using the NIST library with a probability factor ≥ 60%, including hydrocarbons, alcohols, carbonyl compounds, esters, halogen compounds, ethers, nitrogen and/or sulfur compounds and carboxylic acids. Based on the intensity of the relevant chromatographic peaks, the bipolar fibers DVB/CAR/PDMS and CAR/PDMS provided the most efficient extraction of low molecular weight sulfur compounds, considered the main causes of the nauseating smell of these sponges. In addition, these two fibers allowed us to highlight the differences between the two species, as shown in Table 1, which reports the sulfur compounds identified in *Ircinia variabilis* (*Iv*) and *Sarcotragus spinosulus* (*Ss*) samples.

The classes of molecules of the volatile profile of the sponges, characterized using the HS approach, were also detected by DI-SPME-GC-MS. In addition, the DI approach permitted the determination of indole and biogenic amines. Among the fibers tested, the PA coating proved to be more effective for the extraction of the target compounds providing the most intense chromatographic signals and was used for the prosecution of the work.

As far as indole determination is concerned, a linear calibration curve was constructed over the investigated concentration range, which was described by the following equation: y = 1.8 × 10^4^ + 40, with correlation coefficient of 0.9996 and intercept not significantly different from zero at 95% confidence level. The obtained within-day and between-days RSD% values were 4 and 7, respectively, and were not concentration dependent (according to a *t*-test). LOD and LOQ were 0.01 and 0.03 µg/mL, respectively. The estimated indole concentrations in *Ss* specimens were between 0.05 ± 0.01 and 0.48 ± 0.13 µg/g (average 0.28 ± 0.21 µg/g, *n* = 9), whereas it was always at not detectable levels in all *Iv* specimens analyzed. The obtained percentage recovery was 98 ± 2% for all samples.

**Figure 1:**
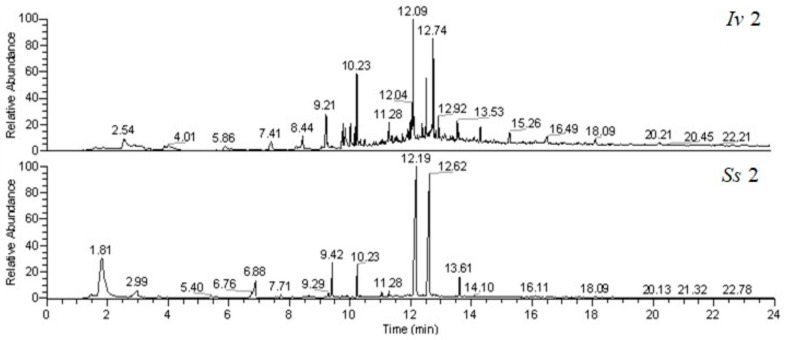
DI-SPME-GC-MS chromatograms, acquired in Total Ion Current (TIC) mode, related to *Ircinia variabilis* (*Iv*, specimen number 2) and *Sarcotragus spinosulus* (*Ss*, specimen number 2), subjected to extraction with a PA fiber after the IBCF addition.

As for the determination of the BAs released by the sponges, Figure 1 shows two typical GC-MS chromatograms related to *Iv* and *Ss* samples subjected to DI-SPME extraction after derivatization with isobutyl chloroformate (IBCF). As evident, the chromatographic profiles for the two species are different and the BAs were identified by comparing both the retention times and the mass spectra of standard solutions analyzed under the same conditions.

Table 2 shows the list of IBCF derivates of BAs determined in the analyzed sponge samples, listed in elution order, the main *m*/*z* ions present in the relative mass spectra, and the relative intensities. Calibration curves were constructed in selected ion monitoring (SIM) acquisition mode for the biogenic amine identified, which was linear and in the range 0.01–1 µg/mL, with correlation coefficients ranging from 0.9951 to 0.9995 (Table 3) and intercepts not significantly different from zero at 95% confidence level.

The obtained within-day and between-days RSD% values were in the ranges of 3.6–6.2 and 7.2–14.3, respectively, and were not concentration dependent (according to a *t*-test). LODs (S/N = 3) and LOQs (S/N = 10) ranged from 0.03 to 0.95 ng/mL and from 0.1 to 3.16 ng/mL, respectively. The validation parameters obtained in the work have been resumed in Table 3.

The average BAs concentrations estimated in both sponges are listed in Table 4. The accuracy of the determinations was assessed by the assessment of the recoveries, whose levels ranged from 85 to 99% (Table 3).

## 3. Discussion

It is known from the literature [19,29,30,31] that in marine organisms the synthesis of toxins with a protective function is usually associated with the production of unpleasant tastes and odors, interpretable in the context of chemical signaling systems in the aquatic environment. In this scenario, we have focused on the sponges *Ircinia variabilis* (*Iv*) and *Sarcotragus spinosulus* (*Ss*), widespread and rather abundant in the Mediterranean. They both belong to the family Irciniidae, which includes species that typically produce a nauseating smell responsible for the family name, given that in Latin “hircus” means goat.

Our investigations were aimed at identifying the pattern of molecules presumably responsible for the sponges’ odor; specifically sulfur compounds, indole, and biogenic amines (BAs). For this purpose, solid phase microextraction (SPME) has been chosen as the extraction procedure, since it represents a faster and cheaper alternative to traditional methods and is solvent-free [28,32]. Primarily, four diverse coated commercial fibers, differing in polarity and composition, were tested for the collection of volatile organic compounds (VOCs) from the headspace (HS) of a hermetically sealed vial containing the specimens of *Iv* and *Ss* collected from the southern Italian coasts.

Overall, the results of this study allowed us to assert that SPME-GC-MS was an effective technique for sampling the molecules released by sponges, including those responsible for their smell. The SPME fiber coating affected the extraction profile acquired in the chromatogram.

Comparing the HS-SPME-GC-MS profiles obtained using the same type of fiber with specimens of *Iv* and *Ss*, no differences were highlighted, except with bipolar fibers, the most suitable for extracting low molecular weight sulfur compounds. Indeed, while dimethyl sulfide was produced by both species, methyl isothiocyanate and dimethyl trisulfide were only detectable in the profiles from *Ss* (Table 1), suggesting a different ability to produce volatile sulfur compounds by *Iv* and *Ss*. As regards the possible role played by sulfur compounds in the sponge, their involvement in the chemical defense has been proposed for *Ircinia felix* from the Caribbean [17]. Indeed, Duque et al. submitted sulfur compounds extracted from the sponge to antimicrobial tests, showing an activity against the pathogens *Pseudomonas aeruginosa*, *Micrococcus luteus*, and *Staphylococcus* *aureus* [17]. Conversely, a palatability test performed on teleost reef fish allowed researchers to reject the hypothesis of a deterrent effect on predators [21]. Furthermore, a possible antifouling function of sulfur compounds, already proposed in the past by Pawlik et al. [21], cannot yet be ruled out.

DI-SPME-GC-MS analysis allowed us to identify indole in sponge release solutions, by means of polyacrylate (PA) fibers, the best able to extract this compound. Another interesting difference between the species studied is the presence of indole only in the pattern of molecules secreted by *Ss*, while the levels of this chemical were undetectable in *Iv*. The presence of indole could be responsible for the more intense bad odor emanating from *Ss* compared to *Iv*. Indole is originated from the amino acid tryptophan by the action of bacteria and seems to be involved in various aspects of bacterial life [33]. Other chemicals produced by the microbial metabolism having unpleasant odor are some BAs [34]. BAs are naturally formed from decarboxylation of amino acids, or their precursor. Specifically, in this study, compounds belonging to the better-known decarboxylation systems, such as histidine/histamine, lysine/cadaverine, and ornithine/putrescine, have been identified and quantified [35]. The chromatograms acquired for the two species showed very different profiles (Figure 1). Through careful inspection of the total ionic current (TIC) chromatograms and mass spectra of all detectable peaks (S/N ≥ 3), eight of the sixteen BAs have been identified that can be efficiently extracted with the method employed [36]. The correctness of the attributions was confirmed by SPME analysis of standard solutions. The parameters relating to the validation of the method listed in Table 3 are in good agreement with the literature [36]. Furthermore, the accuracy of the determinations, evaluated by recovery studies, is adequate for the correctness of the estimates.

Once again, our results showed that the levels of BAs were always much higher in *Ss* than in *Iv* specimens, with isobutylamine, propylamine, and tryptamine detected only in the former species. BAs are included among the so-called “small molecule transmitters”, that have been shown to function in the contraction behavior of sponges, being produced by the sponge itself or by the sponges’ bacterial symbionts [37]. The role of histamine still remains unclear, although recent studies [38] hypothesize that it may intervene in the regulation of inflammatory processes in deuterostomes, and this should further stimulate research interest in the physiology of sponges.

Until now, no information on VOCs produced by *Ss* was available, apart from some data in the gray literature [22] referring to Western Mediterranean populations. Furthermore, the presence of indole and BAs had never been detected before.

On the whole, the greater variety of low molecular weight sulfur compounds and BAs identified by the present study in *Ss* compared to *Iv* seems to be related to the emission of a more intense unpleasant odor. These results call for future bioecological investigations, which may highlight possible differences in the ability of sympatric populations of the two species to defend themselves from external attacks by predators or pathogenic microorganisms. Further interesting roles in which these compounds could be involved, that needs to be clarified, are related to space competition response and prevention of fouling covering. It has been indicated that sponges subjected to stress conditions prefer to invest in cell repair rather than in the production of toxic molecules [39]. This could explain the presence of a remarkable fouling community on reared sponge specimens, as reported by Giangrande et al. [13], contrarily to what usually happens in wild specimens.

Since the fundamental role played by sponge symbionts is the synthesis of bioactive compounds [7,40], these results point out that differences exist in the microbiota colonizing *Iv* and *Ss*. Indeed, these species, like all the members of the family Irciniidae, are classified as high microbial abundance sponges, whose associated microorganisms can perform a wide range of functions of ecological interest. For instance, they can modulate the growth of cells of their host or that of its competitors by producing inhibitory growth signals [41]. Future studies would be necessary to verify the ecological role of the molecules identified in this work.

## 4. Materials and Methods

### 4.1. Sponge Collection

Samples were collected from the abundant and widespread sponge community of the Mar Grande of Taranto (Northern Ionian Sea, 40°25’45.94’’ N, 17°13’17.72’’ E), on rocky substrates at a depth of 7 m, cutting the upper portion of 3 donor specimens of each of the species *Ircinia variabilis* (*Iv*) and *Sarcotragus spinosulus* (*Ss*) (Dictyoceratida, Irciniidae). The specimens selected for sampling were all massive, with a diameter ranging between 10 and 15 cm. Their basal portion was left in place to allow regeneration. After collection, the samples were immediately weighed and smelled (Table 5) before being stored, protected from light, at a temperature of −20 °C.

The identification of collected samples was confirmed in the laboratory by observing, in light microscopy, sections of the sponge’s skeleton prepared according to standard taxonomic techniques.

### 4.2. Chemicals

All standards (indole, eight biogenic amines (BAs), namely cadaverine hydrochloride, histamine dihydrochloride, isobutylamine, isopentylamine, propylamine hydrochloride, 2-phenylethylamine, propylamine hydrochloride, putrescine dihydrochloride, and tryptamine hydrochloride) were purchased from Sigma-Aldrich (Milan, Italy) and were ≥99% pure. Isobutyl chloroformate (IBCF, Sigma-Aldrich, Milan, Italy) was used as derivatizing agent.

Stock solutions (1 mg/mL) were prepared in sterile-filtered ultrapure water (Sigma-Aldrich) and stored in a refrigerator at 4 °C. Working solutions were made by diluting stock solutions with sterile-filtered ultrapure water.

### 4.3. Solid Phase Microextraction (SPME) Fibers and Gas Chromatography-Mass Spectrometry (GC-MS) System

The SPME device, including a manual holder and four different types of fiber coatings (carbowax-polyethylene glycol (PEG) 60 μm, polyacrylate (PA) 85 μm, divinylbenzene/carboxen/polydimethylsiloxane (DVB/CAR/PDMS) 50/30 μm, and carboxen/polydimethylsiloxane (CAR/PDMS) 75 μm) were obtained from Supelco (Sigma-Aldrich). Fibers were conditioned in the GC injector as suggested by the supplier.

The GC-MS system was a Finnigan TRACE GC ultra-gas chromatograph (Thermo Fisher Scientific, Waltham, MA, USA) equipped with a split/splitless injector, interfaced to an ion trap MS (Finnigan Polaris Q, Thermo Fisher Scientific). The capillary column was a Supelco SPB-5 fused silica (30 m, 0.25 µm i.d., 0.25 µm film thickness) with helium (purity > 98%, Rivoira, Bari, Italy) as carrier gas (flow rate 1 mL/min).

The temperature of the transfer line was 220 °C, while the injector (splitless mode) was kept at 200 °C (headspace solid phase microextraction, HS-SPME) and 250 °C (direct immersion solid phase microextraction, DI-SPME). The oven temperature program was: 40 °C held for 5 min; ramp 1: 12 °C/min from 40 °C to 160 °C; 160 °C held for 1 min; ramp 2: 5 °C/min from 160 °C to 200 °C; 200 °C held for 5 min; ramp 3: 10 °C/min from 200 °C to 220 °C; 220 °C held for 1 min. The mass spectrometer was operated in the electron impact positive ion mode (EI+) with the ion source temperature set at 250 °C. The electron energy was 70 eV and the filament current 150 µA. Total ion current (TIC, *m*/*z* range 30–300) or selected ion monitoring (SIM, the *m*/*z* ions useful for the determination are listed in Table 2) acquisition modes were used. The National Institute of Standards and Technology (NIST) library allowed us to identify the revealed molecules.

### 4.4. SPME-GC-MS Experimental Conditions

#### 4.4.1. Volatile Organic Compounds (VOCs) Analyses

Each sponge specimen was shredded, a portion (1 g) taken, and put in a 7.5 mL amber vial. The vial was sealed with hole caps and PTFE/silicon septa (Sigma-Aldrich) and immersed for 30 min in a thermostatic bath at 37 °C, prior to VOCs extraction. The SPME fiber was placed into the headspace 1 cm above the solid sample and exposed for 15 min. Next, the fiber was thermally desorbed in the GC injector for 2 min for the GC-MS analysis (TIC acquisition mode).

#### 4.4.2. Indole and Biogenic Amines (BAs) Analyses

Sponge samples (1 g) were weighed directly into 4.0 mL amber vials (Sigma Aldrich), the vial filled with a 15% NaCl sterile solution in ultra-pure water (3.0 mL), sealed with drillable caps and put in a thermostatic bath at 37 °C for 4 h. Then, 0.5 mL were taken by piercing the silicone septum with the needle of a Hamilton Microliter syringe (Supelco) and transferred into a sealed 1.7 mL clear glass vial, containing 1.0 mL of a sterile 15% NaCl solution and a PTFE Teflon coated magnetic stir bar (4 × 10 mm) (Sigma-Aldrich). The pH of the resulting solution, in accordance with [36], was adjusted to 12 with 4 N NaOH (3.8 µL), added to the sealed vials with a syringe, and subjected to DI-SPME (polyacrylate coating). Eventually, fibers were carefully removed by the vials and inserted directly into the GC injector for 2 min for the GC-MS analysis (TIC acquisition mode for indole, SIM for BAs).

For BAs extraction, the derivatizing agent IBCF (7.5 µL) was added immediately after NaOH addition [36]. The vial was immediately shaken manually for 2 min at room temperature and a polyacrilate fiber was exposed to the solution for 40 min.

Calibration curve for indole was prepared adding blank samples (without sponge fragments, only 15% NaCl sterile solution) with 1 mL of working solutions in the concentration range 0.012–12 µg/mL, corresponding to 0.003–3 µg/mL in the test tube. The within-day (*n* = 3) and between-days (*n* = 3 over 5 days) percentage relative standard deviations (RSD %) were calculated at 0.01, 0.05, and 1 µg/mL. Limits of detection (LOD) and quantitation (LOQ) were calculated at a signal-to-noise (S/N) ratio of 3 and 10, respectively. To estimate recovery, indole was added to each specimen in order to have twice the estimated concentration, or the LOD level, and then the ratio between the peak area in the spiked sample and a standard solution at the same concentration was calculated.

Calibration curves for BAs were prepared adding six blank samples with variable amounts of working solutions in the concentration range 0.04–4 µg/mL, corresponding to 0.01–1 µg/mL in the test tube.

The within-day (*n* = 3) and between-days (*n* = 3 over 5 days) percentage relative standard deviations (RSD %) were calculated at 0.05, 0.25, and 1 µg/mL. LODs and LOQs were calculated as described above. To estimate recovery, mix standard solutions containing the analytes at twice the estimated concentration, or the LOD level, were added to each sample, and then the ratio between the areas of the peaks was calculated as described above.

To eliminate carry over, fibers were always subjected to a second thermal desorption after the chromatographic runs. All experiments were performed in triplicate and blank samples (vials without sponge fragments) were randomly acquired between different samples to exclude fiber signals.

### 4.5. Statistics

The statistical significance was evaluated by two-tailed Student’s *t*-test. Results were considered statistically significant at *p* < 0.05.

## 5. Conclusions

Some marine sponges, particularly those belonging to the genera *Ircinia* and *Sarcotragus*, are known for their unpleasant smell, which becomes much more intense when they are cut. In the present study, thanks to the development of an experimental method for the detection of sulfur compounds, indole, and some BAs, that combines HS and DI-SPME with GC-MS, several volatile compounds in the headspace and molecules released in an aqueous medium by two Mediterranean Irciniidae species were identified. A different composition in sulfur compounds was detected between volatile compounds released by *Ircinia variabilis* (*Iv*) and *Sarcotragus spinosulus* (*Ss*), suggesting a different ability of the two species in producing such chemicals. Moreover, the presence of indole was detectable only in the pattern of molecules secreted by *Ss* and, once again, higher levels of BAs were found in *Ss* than in *Iv* specimens, with isobutylamine, propylamine, and tryptamine detected only in *Ss*. The greater variety of low molecular weight sulfur compounds, indole, and BAs identified by the present study in *Ss* compared to *Iv* seem to be related to the emission of a more intense unpleasant smell by this sponge.

## Figures and Tables

**Table 1:** Volatile sulfur compounds extracted from *Ircinia variabilis* (*Iv*) and *Sarcotragus spinosulus* (*Ss*) by the headspace approach using different fibers, and identified by NIST library. RT = Retention Time; MF = Match Factor; P (%) = Probability Factor (%).

RT (min)	Compound	MF	P (%)	SPME Fiber Coating	
				CAR/PDMS	DVB/CAR/PDMS
2.01 ± 0.01	dimethyl sulfide	915	70.1	*Iv*/*Ss*	*Iv*/*Ss*
4.94 ± 0.02	methyl isothiocyanate	875	82.5	*Ss*	*Ss*
10.90 ± 0.03	dimethyl trisulfide	869	91.9		*Ss*

**Table 2:** IBCF derivates of BAs determined in sponge samples, reported in order of increasing retention time, their *m*/*z* ions, and relative intensities (fragments useful for the determination in SIM acquisition mode are shown in bold).

RT (min)	*m*/*z* Ions (Relative Intensities)	Compound
4.79 ± 0.02	**104** (74) **160** (45) 130 (34) 86 (25)	propylamine
5.40 ± 0.06	**130** (66) **118** (34) 100 (12) 173 (5) 158 (3)	isobutylamine
6.76 ± 0.07	**132** (100) 130 (98) **118** (36) 114 (29) 187 (15)	isopentylamine
10.23 ± 0.09	**104** (100) 130 (79) **91** (76) 221 (30) 148 (18)	2-phenylethylamine
12.19 ± 0.06	**170** (100) **130** (63) 288 (10)	putrescine
**12.52 ± 0.09**	**84** (85) **130** (80) 129 (73) 302 (2)	cadaverine
**13.61 ± 0.06**	**130** (100) **143** (59) 260 (19) 187 (4)	tryptamine
**14.58 ± 0.07**	**194** (100) **138** (25) 238 (16)	histamine

**Table 3:** Equations, coefficient of correlations, precision and limits of BAs with SPME-GC-MS.

Compound	Equation	R^2^	Within-Day (% RSD)	Between-Days (% RSD)	LOD (ng/mL)	LOQ (ng/mL)
2-phenylethylamine	y = 3.3 × 10^3^x + 28	0.9988	3.6	7.3	0.03	0.10
cadaverine	y = 1.4 × 10^3^x − 33	0.9981	5.8	8.1	0.12	0.40
histamine	y = 0.2 × 10^2^x − 8	0.9988	6.2	9.9	0.17	0.57
isobutylamine	y = 0.9 × 10^3^x − 98	0.9987	3.7	10.3	0.95	3.16
isopentylamine	y = 0.8 × 10^3^x + 24	0.9984	4.3	7.2	0.06	0.19
propylamine	y = 1.7 × 10^3^x + 96	0.9995	6.1	14.3	0.21	0.70
putrescine	y = 8.0 × 10^3^x − 94	0.9981	4.5	11.4	0.03	0.10
tryptamine	y = 3.1 × 10^3^x + 22	0.9951	4.6	11.4	0.07	0.23

**Table 4:** BAs average concentration and recoveries for spiked sponge samples (*Sarcotragus spinosulus, Ss*; *Ircinia variabilis*, *Iv*).

Compound	*Ss* (µg/g)	% Rec (*n* = 6)	*Iv* (µg/g)	% Rec (*n* = 9)
2-phenylethylamine	3.56 ± 1.30	99 ± 2	0.65 ± 026	85 ± 3
cadaverine	41.27 ± 20.30	98 ± 3	0.82 ± 0.32	87 ± 4
histamine	2.35 ± 0.98	96 ± 3	1.54 ± 0.89	89 ± 5
isobutylamine	3.22 ± 1.42	99 ± 1		
isopentylamine	10.09 ± 3.09	93 ± 4	0.05 ± 0.03	88 ± 3
propylamine	0.17 ± 0.09	92 ± 3		
putrescine	3.59 ± 1.01	85 ± 3	0.10 ± 0.05	88 ± 3
tryptamine	7.91 ± 2.56	85 ± 3		

**Table 5:** Wet weight and intensity of the odor emanating from the sampled specimens of *Ircinia variabilis* (*Iv*) and *Sarcotragus spinosulus* (*Ss*).

Species	Specimens	Weight (g)	Bad Smell
*Iv*	1	40.62	++
*Iv*	2	61.68	++
*Iv*	3	50.79	++
*Ss*	1	63.59	+++
*Ss*	2	63.16	+++
*Ss*	3	39.74	+++

## Data Availability

Data, associated metadata, and calculation tools are available from the corresponding author.

## Supplementary Materials

The following are available online at https://www.mdpi.com/article/10.3390/md19120711/s1, Figure S1: HS-SPME-GC-MS chromatograms, acquired in Total Ion Current (TIC) mode, related to *Ircinia variabilis* (*Iv*) and *Sarcotragus spinosulus* (*Ss*), subjected to extraction with the four selected fibers.

## References

[1] Van Soest R.W.M., Boury-Esnault N., Vacelet J., Dohrmann M., Erpenbeck D., de Voogd N.J., Santodomingo N., Vanhoorne B., Kelly M., Hooper J.N.A. (2012). Global diversity of sponges (Porifera). PLoS ONE.

[2] Webster N.S., Taylor M.W. (2012). Marine sponges and their microbial symbionts: Love and other relationships. Environ. Microbiol..

[3] Pita L., Rix L., Slaby B.M., Franke A., Hentschel U. (2018). The sponge holobiont in a changing ocean: From microbes to ecosystems. Microbiome.

[4] Genta-Jouve G., Cachet N., Oberhänsli F., Noyer C., Teyssié J.L., Thomas O.P., Lacoue-Labarthe T. (2012). Comparative bioaccumulation kinetics of trace elements in Mediterranean marine sponges. Chemosphere.

[5] Rohde S., Nietzer S., Schupp P.J. (2015). Prevalence and mechanisms of dynamic chemical defenses in tropical sponges. PLoS ONE.

[6] Rust M., Helfrich E.J.N., Freeman M.F., Nanudorn P., Field C.M., Rückert C., Kündig T., Page M.J., Webb V.L., Kalinowski J. (2020). A multiproducer microbiome generates chemical diversity in the marine sponge *Mycale hentscheli*. Proc. Natl. Acad. Sci. USA.

[7] Lee Y.-J., Cho Y., Tran H.N.K. (2021). Secondary metabolites from the marine sponges of the genus *Petrosia*: A literature review of 43 years of research. Mar. Drugs.

[8] Mehbub M.F., Perkins M.V., Zhang W., Franco C.M.M. (2016). New marine natural products from sponges (Porifera) of the order Dictyoceratida (2001 to 2012); a promising source for drug discovery, exploration and future prospects. Biotechnol. Adv..

[9] Abdelaleem E.R., Samy M.N., Desoukey S.Y., Liu M., Quinn R.J., Abdelmohsen U.R. (2020). Marine natural products from sponges (Porifera) of the order Dictyoceratida (2013 to 2019); a promising source for drug discovery. RSC Adv..

[10] Mercurio M., Longo C., Nonnis Marzano C., Scalera Liaci L., Corriero G. (2003). L’allevamento di spugne commerciali nella Riserva Naturale Marina ‘Isola di Ustica’. Biol. Mar. Mediterr..

[11] Corriero G., Longo C., Mercurio M., Nonnis Marzano C., Lembo G., Spedicato M.T. (2004). Rearing performance of *Spongia officinalis* on suspended ropes off the Southern Italian Coast (Central Mediterranean Sea). Aquaculture.

[12] Baldacconi R., Cardone F., Longo C., Mercurio M., Nonnis Marzano C., Gaino E., Corriero G. (2010). Transplantation *of Spongia officinalis* L. (Porifera, Demospongiae): A technical approach for restocking this endangered species. Mar. Ecol. Evol. Persp..

[13] Giangrande A., Pierri C., Arduini D., Borghese J., Licciano M., Trani R., Corriero G., Basile G., Cecere E., Petrocelli A. (2020). An innovative IMTA system: Polychaetes, sponges and macroalgae co-cultured in a Southern Italian in-shore mariculture plant (Ionian Sea). J. Mar. Sci. Eng..

[14] Longo C., Scrascia M., Trani R., Pierri C., Cariglia A., Cariglia F., Cariglia M. Assesment of sponge mariculture potential in polyculture system in Manfredonia Gulf toward the IMTA implementation. Proceedings of the Aquafarm Novelfarm 2020.

[15] Christophersen C., Anthoni U., Nielsen P.H., Jacobsen N., Tendal O.S. (1989). Source of a nauseating stench from the marine sponge, *Halichondria panicea*, collected at Clever Bank in the North Sea. Biochem. Syst. Ecol..

[16] Roussis V., Mazomenos B.E., Vayas K., Harvala C. (1995). Comparative study on the volatile metabolites of two marine sponge species of the genus Plakortis. J. Essent. Oil Res..

[17] Duque C., Bonilla A., Bautista E., Zea S. (2001). Exudation of low molecular weight compounds (thiobismethane, methyl isocyanide, and methyl isothiocyanate) as a possible chemical defense mechanism in the marine sponge *Ircinia felix*. Biochem. Syst. Ecol..

[18] De Rosa S., Kamenarska Z., Seizova K., Iodice C., Petrova A., Nedelcheva D., Stefanov K., Popev S. (2008). Volatile and polar compounds from *Geodia cydonium* and two *Tedania* species. Bulg. Chem. Commun..

[19] Mollo E., Fontana A., Roussis V., Polese G., Amodeo P., Ghiselin M.T. (2014). Sensing marine biomolecules: Smell, taste, and the evolutionary transition from aquatic to terrestrial life. Front. Chem..

[20] Mollo E., Garson M.J., Polese G., Amodeo P., Ghiselin M.T. (2017). Taste and smell in aquatic and terrestrial environments. Nat. Prod. Rep..

[21] Pawlik J.R., McFall G., Zea S. (2002). Does the odor from sponges of the genus *Ircinia* protect them from fish predators?. J. Chem. Ecol..

[22] Abed C. (2011). Spongiaires Irciniidae de Méditerranée: Chimiotaxonomie, Métabolites Volatils et Bio-Indicateurs de Pollution par les Eléments Traces Métalliques. Ph.D. Thesis.

[23] da Frota M.J.L.C., da Silva R.B., Mothes B., Henriques A.T., Moreira J.C.F. (2012). Current status on natural products with antitumor activity from Brazilian marine sponges. Curr. Pharm. Biotechnol..

[24] Longeon A., Copp B.R., Quévrain E., Roué M., Kientz B., Cresteil T., Petek S., Debitus C., Bourguet-Kondracki M.-L. (2011). Bioactive indole derivatives from the South Pacific marine sponges *Rhopaloeides odorabile* and *Hyrtios* sp. Mar. Drugs.

[25] Hanif N., Yamada K., Kitamura M., Kawazoe Y., de Voogd N.J., Uemura D. (2015). New Indole Alkaloids from the Sponge *Plakortis* sp. Chem. Nat. Compd..

[26] Erdag D., Merhan O., Yildiz B. (2018). Biochemical and pharmacological properties of biogenic amines. Biog. Amin..

[27] Doeun D., Davaatseren M., Chung M.-S. (2017). Biogenic amines in foods. Food Sci. Biotechnol..

[28] De Vietro N., Aresta A.M., Picciariello A., Rotelli M.T., Zambonin C. (2021). Determination of VOCs in Surgical Resected Tissues from Colorectal Cancer Patients by Solid Phase Microextraction Coupled to Gas Chromatography–Mass Spectrometry. Appl. Sci..

[29] Anjum K., Abbas S.Q., Shah S.A.A., Akhter N., Batool S., Hassan S.S.U. (2016). Marine Sponges as a Drug Treasure. Biomol. Ther..

[30] Mehbub M.F., Lei J., Franco C., Zhang W. (2014). Marine sponge derived natural products between 2001 and 2010: Trends and opportunities for discovery of bioactives. Mar. Drugs.

[31] Thakur N.L., Singh A. (2016). Chemical ecology of marine sponges. Marine Sponges: Chemicobiological and Biomedical Applications.

[32] Misharina T.A., Terenina M.b., Krikunova N.I. (2017). Determination of volatile organic compounds by solid-phase microextraction. Appl. Biochem. Microbiol..

[33] Hu M., Zhang C., Mu Y., Shen Q., Feng Y. (2010). Indole affects biofilm formation in bacteria. Indian J. Microbiol..

[34] Al Bulushi I., Poole S., Deeth H., Dykes G. (2018). Evaluation the spoilage and biogenic amines formation potential of marine Gram-positive bacteria. Int. Food Res. J..

[35] Al Bulushi I., Poole S., Deeth H.C., Dykes G.A. (2009). Biogenic amines in fish: Roles in intoxication, spoilage, and nitrosamine formation—A review. Crit. Rev. Food Sci. Nutr..

[36] Papageorgiou M., Lambropoulou D., Morrison C., Namieśnik J., Płotka-Wasylka J. (2018). Direct solid phase microextraction combined with gas chromatography–Mass spectrometry for the determination of biogenic amines in wine. Talanta.

[37] Leys S.P. (2015). Elements of a ‘nervous system’ in sponges. J. Exp. Biol..

[38] D’Aniello E., Paganos P., Anishchenko E., D’Aniello S., Arnone M.I. (2020). Comparative Neurobiology of Biogenic Amines in Animal Models in Deuterostomes. Front. Ecol. Evol..

[39] Agell G., Uriz M., Cebrian E., Martí R. (2001). Does stress protein induction by copper modify natural toxicity in sponges?. Environ. Toxicol. Chem. Int. J..

[40] Stévenne C., Micha M., Plumier J.-C., Roberty S. (2021). Corals and Sponges Under the Light of the Holobiont Concept: How Microbiomes Underpin Our Understanding of Marine Ecosystems. Front. Mar. Sci..

[41] Hardoim C.C.P., Costa R. (2014). Microbial communities and bioactive compounds in marine sponges of the family Irciniidae—A review. Mar. Drugs.

