# Supplementary material for: Volatile Organic Compounds, Indole, and Biogenic Amines Assessment in Two Mediterranean Irciniidae (Porifera, Demospongiae)
Source: Mar Drugs. 2021 Dec 17;19(12):711. doi: 10.3390/md19120711 (PMC8706640; doi:10.3390/md19120711)
Supplement: Supplementary file 1 [file marinedrugs-19-00711-s001.zip › marinedrugs-1497085-supplementary.pdf]

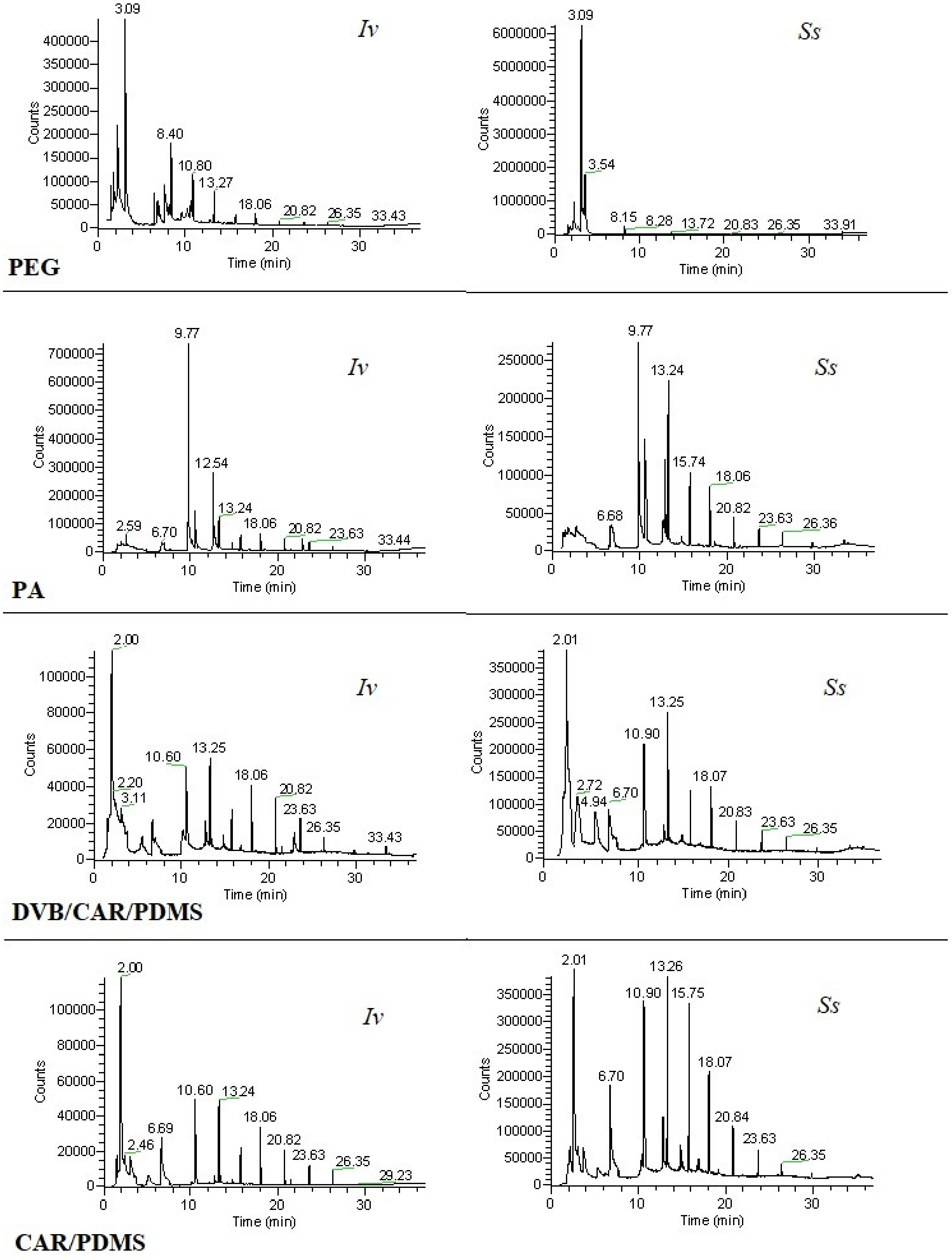

Figure S1. HS-SPME-GC-MS chromatograms, acquired in Total Ion Current (TIC) mode, related to *Ircinia variabilis* (*Iv*) and *Sarcotragus spinosulus* (*Ss*), subjected to extraction with the four selected fibers.
